# Proteolytic Activity of the Paracaspase MALT1 Is Involved in Epithelial Restitution and Mucosal Healing

**DOI:** 10.3390/ijms24087402

**Published:** 2023-04-17

**Authors:** Leonie Wittner, Lukas Wagener, Jakob J. Wiese, Iris Stolzer, Susanne M. Krug, Elisabeth Naschberger, Rene Jackstadt, Rudi Beyaert, Raja Atreya, Anja A. Kühl, Gregor Sturm, Miguel Gonzalez-Acera, Jay V. Patankar, Christoph Becker, Britta Siegmund, Zlatko Trajanoski, Beate Winner, Markus F. Neurath, Michael Schumann, Claudia Günther

**Affiliations:** 1 Department of Medicine 1, Friedrich-Alexander University Erlangen-Nürnberg, 91054 Erlangen, Germany; leonie.wittner@mail.de (L.W.); lukas.wagener@fau.de (L.W.); iris.stolzer@uk-erlangen.de (I.S.); raja.atreya@uk-erlangen.de (R.A.); miguel.gonzalezacera@uk-erlangen.de (M.G.-A.); jay.patankar@uk-erlangen.de (J.V.P.); christoph.becker@uk-erlangen.de (C.B.); markus.neurath@uk-erlangen.de (M.F.N.); 2 Department of Gastroenterology, Rheumatology and Infectious Diseases, Charité-Universitätsmedizin Berlin, Campus Benjamin Franklin, 12203 Berlin, Germany; jakob.wiese@charite.de (J.J.W.); britta.siegmund@charite.de (B.S.); michael.schumann@charite.de (M.S.); 3 Clinical Physiology/Nutritional Medicine, Charité-Universitätsmedizin Berlin, Campus Benjamin Franklin, 12203 Berlin, Germany; susanne.m.krug@charite.de; 4 Division Molecular and Experimental Surgery, Friedrich-Alexander University Erlangen-Nürnberg, 91054 Erlangen, Germany; elisabeth.naschberger@uk-erlangen.de; 5 Cancer Progression and Metastasis Group, German Cancer Research Center (DKFZ), 69120 Heidelberg, Germany; r.jackstadt@hi-stem.de; 6 VIB—UGent Center for Inflammation Research, Department of Biomedical Molecular Biology, Ghent University, 9052 Ghent, Belgium; rudi.beyaert@irc.vib-ugent.be; 7 Deutsches Zentrum Immuntherapie, Friedrich-Alexander University Erlangen-Nürnberg, 91054 Erlangen, Germany; beate.winner@fau.de; 8 IBDome Consortium, 91054 Erlangen, Germany; anja.kuehl@charite.de (A.A.K.); zlatko.trajanoski@i-med.ac.at (Z.T.); 9 IBDome Consortium, 12203 Berlin, Germany; 10 iPATH.Berlin-Core Unit, Charité-Universitätsmedizin Berlin, Campus Benjamin Franklin, 12203 Berlin, Germany; 11 Biocenter, Institute of Bioinformatics, Medical University of Innsbruck, 6020 Innsbruck, Austria; gregor.sturm@i-med.ac.at; 12 Department of Stem Cell Biology, Friedrich-Alexander University Erlangen-Nürnberg, 91054 Erlangen, Germany; 13 Center of Rare Diseases (ZSEER), University Hospital Erlangen, Friedrich-Alexander University Erlangen-Nürnberg, 91054 Erlangen, Germany

**Keywords:** ulcerative colitis, mucosal wound healing, ferroptosis

## Abstract

The paracaspase MALT1 is a crucial regulator of immune responses in various cellular contexts. Recently, there is increasing evidence suggesting that MALT1 might represent a novel key player in mucosal inflammation. However, the molecular mechanisms underlying this process and the targeted cell population remain unclear. In this study, we investigate the role of MALT1 proteolytic activity in the context of mucosal inflammation. We demonstrate a significant enrichment of MALT1 gene and protein expression in colonic epithelial cells of UC patients, as well as in the context of experimental colitis. Mechanistically we demonstrate that MALT1 protease function inhibits ferroptosis, a form of iron-dependent cell death, upstream of NF-κB signaling, which can promote inflammation and tissue damage in IBD. We further show that MALT1 activity contributes to STAT3 signaling, which is essential for the regeneration of the intestinal epithelium after injury. In summary, our data strongly suggests that the protease function of MALT1 plays a critical role in the regulation of immune and inflammatory responses, as well as mucosal healing. Understanding the mechanisms by which MALT1 protease function regulates these processes may offer novel therapeutic targets for the treatment of IBD and other inflammatory diseases.

## 1. Introduction

Ulcerative colitis (UC) is a chronically relapsing inflammatory disease starting in the rectum and extending throughout the colon. Patients suffer from abdominal pain, rectal bleeding, and diarrhea. The underlying etiology is multifactorial and is associated with genetic predisposition, environmental risk factors, imbalanced microbial composition, dysregulated immune responses, and intestinal epithelial barrier dysfunction [1,2]. In this multifactorial setting, the intestinal epithelium is a single epithelial layer that serves as a selective permeable barrier between the intestinal lumen and the underlying immune cell compartment and thus maintains mucosal homeostasis [3]. Deregulated cell death in the intestinal epithelium is a critical driver of barrier dysfunction and subsequent inflammation [4,5,6,7,8]. Among the different types of inflammatory cell death, ferroptosis has been recently described as an important regulated cell death pathway contributing to the pathogenesis of UC. In the intestinal epithelium, ferroptotic cell death is mediated by ER-stress and can be inhibited by phosphorylation of the NF-κB subunit p65 [6].

NF-κB signaling has been well characterized in the context of intestinal inflammation, infection and cancer, where it can exert both detrimental and protective functions. Especially during mucosal wound healing, it has been reported that NF-κB mediates proliferative and pro-survival gene expression and thereby is considered to play an important protective function. Under steady state conditions, the NF-κB subunit p65 mediates anti-apoptotic signaling and preserves intestinal stem cell homeostasis. In the context of epithelial injury, p65 is highly expressed especially at wound edges to induce cell migration and mucosal healing [9]. Accordingly, loss of p65 in the intestinal epithelium is associated with severe mucosal injury and susceptibility to experimental colitis triggered by deregulated intestinal proliferation [10]. Activity of the NF-κB essential modulator (NEMO) is required for NF-κB activation in response to inflammatory stimuli. Intestinal epithelial cell (IEC) specific deletion of NEMO negatively affects epithelial barrier function through excessive activation of TNF-mediated apoptosis in colonocytes associated with severe chronic inflammation [11].

The paracaspase MALT1 (mucosa-associated lymphoid tissue lymphoma translocation protein 1) is an upstream signaling component of NF-κB with high therapeutic potential. Although these data implicate a potential role of MALT1 in orchestrating mucosal immune response, inflammation and tumorigenesis, the role of MALT1 scaffolding and proteolytic activity in the pathogenesis of mucosal inflammation has not been addressed. So far it is known, that various immunoreceptors of the innate and adaptive immune response activate MALT1, which is best characterized in the context of T cell receptor (TCR) and B cell receptor (BCR) mediated lymphocyte development, activation and proliferation [12,13,14]. It is further involved in ITAM- (immunoreceptor tyrosine-based activation motif) coupled natural killer (NK) cell receptor signaling [15] and in non-hematopoietic cells in epidermal growth factor receptor (EGFR) signaling [16] and G protein-coupled receptor (GPCR) signaling [17]. For a long time MALT1 was believed to function as a scaffold protein, providing an assembly platform for NF-κB activation. Besides its scaffold function, MALT1 was described as a paracaspase carrying a proteolytic activity. The protease activity of MALT1 facilitates optimal NF-κB and AP-1 activation by proteolytic cleavage of negative regulators, such as A20, CYLD and RELB. Other MALT1 substrates regulate mRNA stability (Regnase-1, Roquin-1) [18,19,20]. Upon surface receptor activation, CARMA/CARD, BCL10 and MALT1 form the CBM complex serving as a protein assembly platform for NF-κB activation [17,21]. TRAF6 (TNF receptor associated factor 6) and NEMO are further recruited to the CBM complex to induce a NF-κB activation cascade in response to TCR, BCR and GPCR activation [21,22,23]. Mutations in genes belonging to the CBM complex and consequent loss of MALT1 function are associated with human combined immunodeficiency [14,24]. Symptoms of human MALT1 deficiency include severe inflammation of the esophagus accompanied by absence of naïve and memory B cell populations and functional B and T cell defects [14]. In line with this, mice expressing a genetically inactivated MALT1 protease variant display immune defects including a reduction in the regulatory T cell and CD4^+^ T cell compartment. They develop a progressive systemic inflammation early, driven by an altered T cell compartment and accompanied by lymphocyte infiltration in various organs also affecting the gastrointestinal tract [12,25]. Protease mutant mice further display an elevated disease burden in experimental DSS colitis compared to wild-type controls [26]. Accordingly, several lines of evidence indicate that MALT1 protease might play an important role in maintaining mucosal homeostasis and that dysfunction or deregulation contributes to the development and maintenance of intestinal inflammation. 

While MALT1 protease has been proposed as a therapeutic target in acute colitis [27], its role in mucosal healing remains unclear. Disruption of epithelial barrier function and thus loss of intestinal homeostasis is a key feature of inflammatory bowel disease (IBD). Therefore, the recovery process requires thorough wound healing, which is also mediated by proliferative and pro-survival NF-κB signaling. The objective of this study was to identify if and how the MALT1 protease is involved in NF-κB mediated wound healing in response to intestinal epithelial injury.

## 2. Results

### 2.1. Epithelial MALT1 Is Upregulated in Ulcerative Colitis Patients

**Figure 1:**
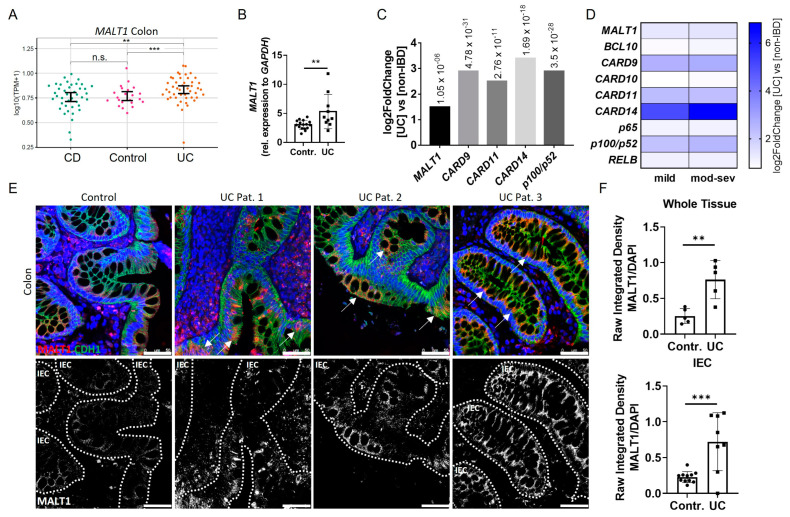
Increased intestinal epithelial *MALT1* expression in UC patients. (**A**) RNA-seq expression levels of *MALT1* in colon tissue samples derived from Crohn’s disease (CD) (n = 41) and ulcerative colitis (UC) (n = 46) patients and controls (n = 22). *p* values are derived from a two-tailed Wilcoxon test. Error bars represent 95% confidence intervals of the mean. (**B**) Quantitative real-time PCR of *MALT1* mRNA expression levels in whole colon biopsies derived from control individuals (n = 16) and UC patients (n = 10). ** *p* < 0.01 in unpaired two-tailed *t*-test, error bars represent mean ± SD, 95% CI. (**C**) RNA-seq expression analysis of significantly altered CBM complex (*MALT1*, *CARD9*, *-11*, *-14*) and NF-κB genes (*p100*/*p52*) in colon biopsies derived from treatment-naïve UC patients (n = 206) and non-IBD controls (n = 20). *p* values are indicated with the fold change. ANOVA for multiple comparisons, error bars represent mean ± SD, 95% CI. (**D**) RNA-seq expression analysis of CBM complex (*MALT1*, *BCL10*, *CARD9–11*, *-14*) and NF-κB (*p65*, *p100*/*p52*, *RELB*) genes in mild (n = 53) and moderate–severe (mod–sev, n = 152) inflamed treatment-naïve UC patients and non-IBD controls (n = 20). (**E**) Representative images of colon cross sections from control individuals and UC patients immunohistochemically stained with antibodies against E-Cadherin (CDH1, green) and MALT1 (red), nuclei were counterstained with Hoechst 33342 (blue) (upper panel). High MALT1 expressing intestinal epithelial areas are indicated with arrows. Representation of MALT1 single channel (white), intestinal epithelial cells (IEC) are indicated with dashed lines (lower panel) (scale bar: 50 µm). (**F**) Quantification of MALT1 expression normalized to Hoechst intensity in whole colon tissue and IECs. n.s. (not significant), ** *p* < 0.01, *** *p* < 0.001 in unpaired two-tailed *t*-test, error bars represent mean ± SD, 95% CI.

In order to investigate the role of MALT1 in the context of chronic intestinal inflammation, we initially analyzed *MALT1* expression patterns in IBD patients. An RNA-seq dataset from colonic tissue of non-IBD, Crohn’s disease (CD) and ulcerative colitis (UC) patients revealed significantly increased *MALT1* gene expression in the colon of UC patients (Figure 1A), which was confirmed by quantification of whole colon RNA lysates derived from a cohort of UC patients and healthy controls (Figure 1B). Similarly, analysis of the PROTECT cohort (GSE109142), an RNA-seq dataset of rectal biopsies derived from newly-diagnosed pediatric UC patients prior to therapeutic intervention [28], revealed a 1.5 fold upregulation of *MALT1* and even stronger upregulation of *CARD9* (2.9 fold change), *CARD11* (2.5 fold change), *CARD14* (3.4 fold change), and p*100*/*p52* (2.9 fold change) (Figure 1C), additional members of the CBM signalosome. Within the CARD family, *CARD14* was particularly upregulated in UC patients and correlated positively with disease severity. Accordingly, moderate-to-severe inflammation was associated with higher *CARD14* expression than mild inflammation. (Figure 1D) We further confirmed an increase in MALT1 on a protein level, which was most prominent in intestinal epithelial cells, as demonstrated by immunofluorescence co-staining with the epithelial cell marker E-cadherin in colonic UC sections (Figure 1E). This increase was also confirmed by quantification of the immunofluorescence signal (Figure 1F). Together, this suggests a potential contribution of epithelial MALT1 to the pathogenesis of UC. 

### 2.2. Malt1 Expression Is Upregulated in Response to Mucosal Injury

**Figure 2:**
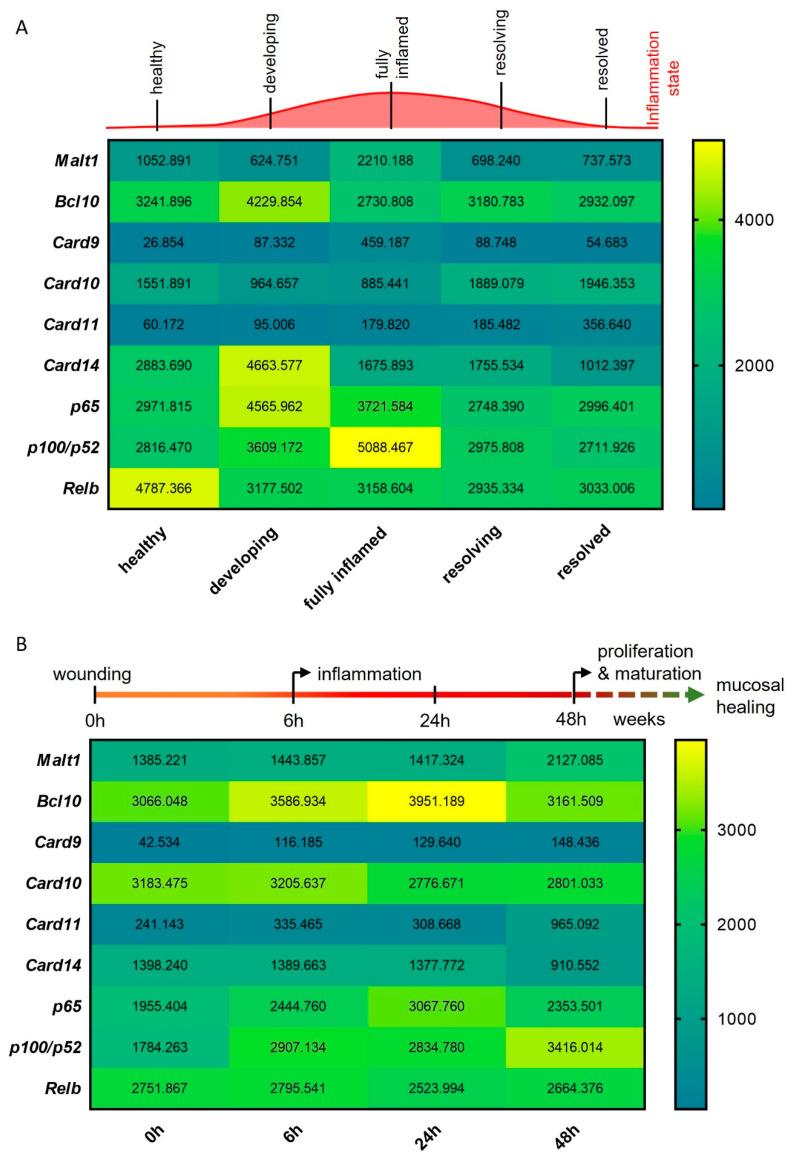
MALT1-mediated signaling is activated during experimental colitis and epithelial damage. (**A**,**B**) RNA-seq expression analyses of CBM complex genes (*Malt1*, *Bcl10*, *Card9–11*, *-14*) and NF-κB subunit genes (*p65*, *p100*/*p52*, *Relb*) during (**A**) a murine DSS colitis time course (n = 3) and (**B**) a murine colon wound healing time course (n = 3).

To study the expression of *Malt1* during the onset, progression and resolution of mucosal inflammation, we investigated *Malt1* expression in an RNA-seq dataset from a murine experimental dextran sodium sulfate (DSS) colitis time course. We detected significantly elevated expression levels in fully inflamed colonic tissue, which subsided during the resolution of the inflammation (Figure 2A). In line with this, *Card9* expression, an essential regulator of intestinal epithelial wound healing in the context of colitis [29] and member of the CBM signalosome, was significantly upregulated during intestinal inflammation. We further observed significantly elevated expression levels of the MALT1 effector proteins *pP100*/*p52* and *Relb* during high inflammation and resolution, respectively (Figure 2A). We additionally compared *Malt1* expression levels in an RNA-seq dataset from a murine wound healing time course. Under these experimental conditions, *Malt1* and *Card11* were significantly upregulated at 48 h after wounding, indicating a potential contribution to epithelial proliferation and mucosal healing. Early upregulation of *Bcl10* and *Card9* further suggests a role for the CBM complex in mucosal wound healing, that potentially induces classical as well as alternative NF-κB signaling as shown by consistent upregulation of *p65* and *p100*/*p52* (Figure 2B).

In summary these data point to a central role of MALT1 in orchestrating inflammatory signaling in response to mucosal injury, potentially promoting NF-κB signaling activation in response to inflammation to foster mucosal healing. 

### 2.3. MALT1 Protease Is Necessary for Cell Proliferation and Viability

**Figure 3:**
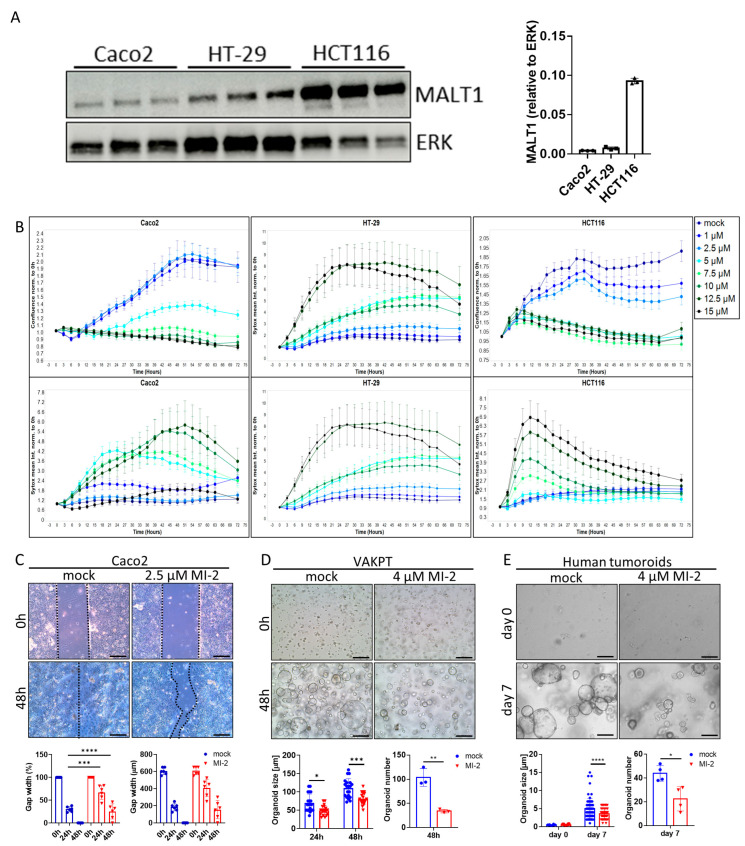
MALT1 proteolytic activity controls cell proliferation and viability. (**A**) Western blot analysis and protein volume quantification of MALT1 protein levels in Caco2, HT-29 and HCT116 cells. ERK was used as a loading control. (**B**) Live cell analysis of Caco2, HT-29 and HCT116 cells stimulated with indicated MI-2 concentrations. Cell confluence (upper panel) and Sytox orange intensity (lower panel) indicate cell growth and cell death over time (hours). (**C**) Scratch assay of Caco2 cells stimulated with 2.5 µM MI-2. Cell growth was monitored for 48 h, gap width was measured every 24 h for quantification (scale bar: 200 µm). *** *p* < 0.001, **** *p* < 0.0001 in ANOVA for multiple comparisons, error bars represent mean ± SD, 95% CI. (**D**,**E**) Tumor organoid formation assays using (**D**) murine tumor organoids (VAKPT) and (**E**) human tumor organoids (CRC patients). Single tumor cells were stimulated with 4 µM MI-2 and monitored over time (scale bar: 200 µm). * *p* < 0.05, ** *p* < 0.01, *** *p* < 0.001, **** *p* < 0.0001 in ANOVA for multiple comparisons and in unpaired two-tailed *t*-test; error bars represent mean ± SD, 95% CI.

The scaffold function of MALT1 has been proposed as a therapeutic target in experimental colitis [27]. To study a specific contribution of the MALT1 protease to mucosal healing, we used the small molecule inhibitor MI-2, targeting specifically the paracaspase domain of MALT1 [30]. The effect of MALT1 protease inhibition on colorectal cancer (CRC) cell proliferation and survival was analyzed in vitro using the cell lines Caco2, HT-29, and HCT116 (Figure 3A). Impaired proliferation was observed in MI-2 dose escalation studies as the inhibitor concentrations increased, and this was accompanied by an increase in the mean Sytox Orange intensities, indicating an increase in cell death (Figure 3B). Thus, high MI-2 concentrations were sufficient to induce cell death in each cell line. As Caco2, HT-29, and HCT116 cells express different MALT1 protein levels (Figure 3A), each cell line displayed cell line-specific dose responses to MALT1 protease inhibition, correlating with the protein level. Accordingly, low MALT1 protein expressing Caco2 cells displayed low sensitivity towards MI-2 treatment. In contrast, HT-29 and HCT116 cells expressing higher MALT1 protein levels likewise showed higher sensitivity towards MI-2 (Figure 3A,B). The necessity of MALT1 proteolytic activity for cell proliferation was further confirmed in a Caco2 wound healing assay, where MI-2 treatment significantly delayed the gap closure at 48 h compared to mock treated cells (Figure 3C). Moreover, we demonstrated that MALT1 protease inhibition affects murine VAKPT (VillinCre, Apc, Kras, P53, Alk5 (TGF-b-signaling)) and human patient-derived tumor (CRC-tumoroids) organoid spheroid-formation. In response to MI-2, fewer organoids arose from single cells, which were also smaller in size than control organoids (Figure 3D,E). Inhibitor specificity was confirmed using murine small intestinal organoids carrying a specific deletion of *Malt1* in intestinal epithelial cells (*Malt1*^ΔIEC^). *Malt1*^ΔIEC^ organoids were protected from MI-2 induced impairment of cell viability (Figure S1A). In line with its important function as NF-κB regulator, we observed a significant decrease in gene expression of the NF-κB target gene *Nos2* (nitric oxide synthase 2) in intestinal organoids in response to MI-2 stimulation (Figure S1B). These data indicate a pivotal role of the MALT1 protease in promoting cell proliferation and viability which may have a beneficial effect on epithelial restitution and mucosal healing in response to intestinal inflammation.

### 2.4. MALT1 Protease Inhibition Causes Ferroptosis In Vitro

**Figure 4:**
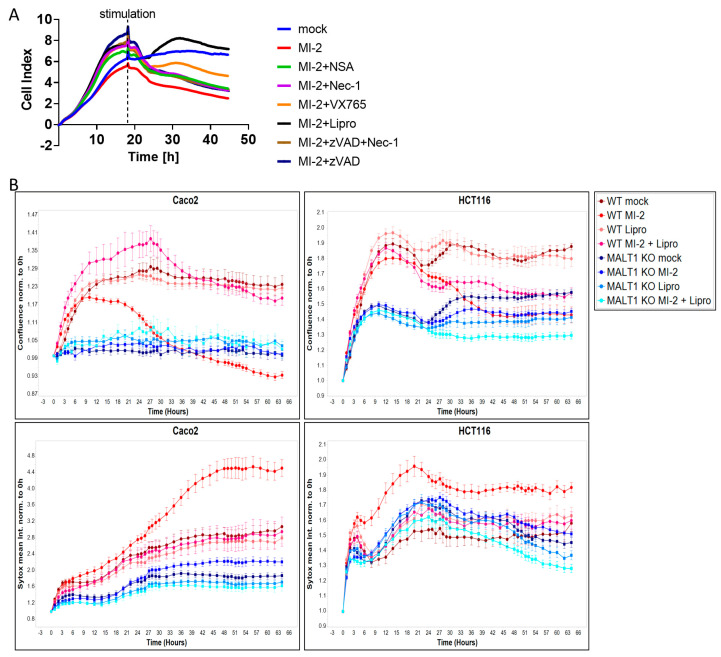
MALT1 protease negatively regulates ferroptosis in vitro. (**A**) Cell death screen of Caco2 cells stimulated with 13.5 µM MI-2, 0.5 µM necrosulfonamide (NSA), 10 µM necrostatin-1 (Nec-1), 20 µM VX-765, 20 µM z-VAD-FMK (zVAD) and 0.2 µM liproxstatin-1 (Lipro). (**B**) Live cell analysis of Caco2 and HCT116 cells stimulated with 13.5 µM MI-2 and 0.2 µM liproxstatin-1 (Lipro). Cell confluence (upper panel) and Sytox green intensity (lower panel) indicate cell growth and cell death over time (hours).

Having shown that MALT1 proteolytic activity promotes epithelial cell proliferation and is necessary for cell viability, we next performed a cell death screen to evaluate the effects of MALT1 inhibition on cell viability and to elucidate which cell death pathway is regulated by MALT1 protease. We found that treatment with MI-2 caused a significant decrease in cell viability and that the addition of the ferroptosis inhibitor liproxstatin-1 [31] was sufficient to rescue Caco2 cells from MI-2 induced cell death (Figure 4A). In contrast none of the other cell death inhibitors targeting apoptosis or regulated necrosis was able to block MI-2 induced toxicity, suggesting that the MALT1 protease is an inhibitor of ferroptosis. As expected, MI-2 failed to induce cell death in Caco2 *MALT1* knockout (KO) cells and HCT116 *MALT1* KO cells, which likewise exposed low Sytox green levels (Figure 4B). In contrast, MI-2 treated wild-type (WT) Caco2 and HCT116 cells exhibited the highest Sytox green intensity and cell death starting around 18 h post stimulation, which was completely rescued by additional liproxstatin-1 treatment (Figure 4B). These data suggest that MALT protease functions upstream of NF-κB to inhibit ferroptosis. 

### 2.5. MALT1 Protease Inhibition Impairs Intestinal Barrier Function In Vitro

**Figure 5:**
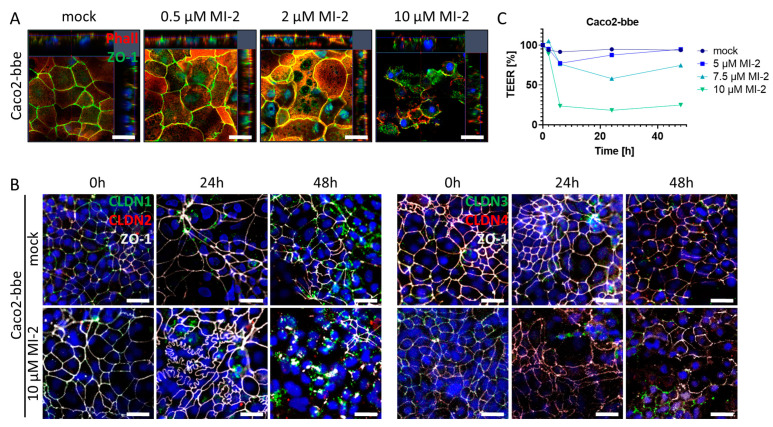
MALT1 proteolytic activity supports epithelial tight junctions. (**A**) Representative images of Caco2-bbe cell monolayers stimulated with indicated MI-2 concentrations 48 h after stimulation immunocytochemically stained with antibody against ZO-1 (green) and phalloidin-594 dye (Phall, red), nuclei were counterstained with Hoechst 33342 (blue) (scale bar: 50 µm). (**B**) Representative images of 10 µM MI-2 stimulated Caco2-bbe cell monolayers over time. Monolayers were immunocytochemically stained with antibodies against ZO-1 (white) and in the left panel claudin-1 (green), claudin-2 (red) and in the right panel claudin-3 (green), claudin-4 (red), nuclei were counterstained with Hoechst 33342 (blue) (scale bar: 50 µm). (**C**) TEER measurement of MI-2 stimulated Caco2-bbe cells.

The intestinal epithelial barrier is a crucial defense mechanism of the gut, which separates the luminal contents from the mucosal immune system. Tight junctions are specialized structures between adjacent epithelial cells that seal the epithelial monolayer and regulate paracellular transport as well as the exchange between environmental factors within the gut lumen and the mucosal immune system located within the lamina propria. Pathophysiological alterations within this structural part of the epithelium can severely compromise mucosal barrier function which may trigger an inflammatory reaction [32]. To investigate the impact of MALT1 proteolytic activity on tight junction biology, we used the Caco2-brush border epithelial (bbe) cell line to establish a single-cell monolayer on a permeable support membrane. The cells were treated with MI-2 at various concentrations. The effect of pharmacological MALT1 protease inhibition on cell–cell contacts was visualized by immunofluorescence staining for the tight junction-associated protein Zonula occludens-1 (ZO-1) using live cell imaging with confocal microscopy (Figure 5A). Already at the lowest inhibitor concentration (0.5 µM), ZO-1 was relocated from the cell–cell border and accumulated within the cytoplasm of the cell. This effect was even more prominent with increasing concentrations of MI-2. Consequently, cells started to die, and the cell layer was disintegrated. Similar, tight junction proteins of the Claudin family were rearranged in Caco2-bbe cells upon MALT1 protease inhibition, leading to cell layer disintegration and cell death (Figure 5B). Rapid and dose-dependent decrease in the trans-epithelial electrical resistance (TEER) in response to MI-2 treatment confirmed the pathophysiological effect of MI-2 on tight junction biology and barrier function (Figure 5C). These data strongly suggest that MALT1 protease activity is important for maintaining the integrity of the intestinal epithelial barrier function, an essential step during mucosal healing. MALT1 inhibition disrupts the localization of the tight junction protein ZO-1 and leads to a loss of tight junction density, resulting in impaired barrier function. In contrast to the suggested therapeutic effect of MI-2 on acute colitis [27], these findings suggest that MALT1 inhibitors may have unintended effects on the intestinal epithelial barrier function.

### 2.6. MALT1 Protease Promotes pSTAT3 Mediated Mucosal Wound Healing in Experimental Colitis

**Figure 6:**
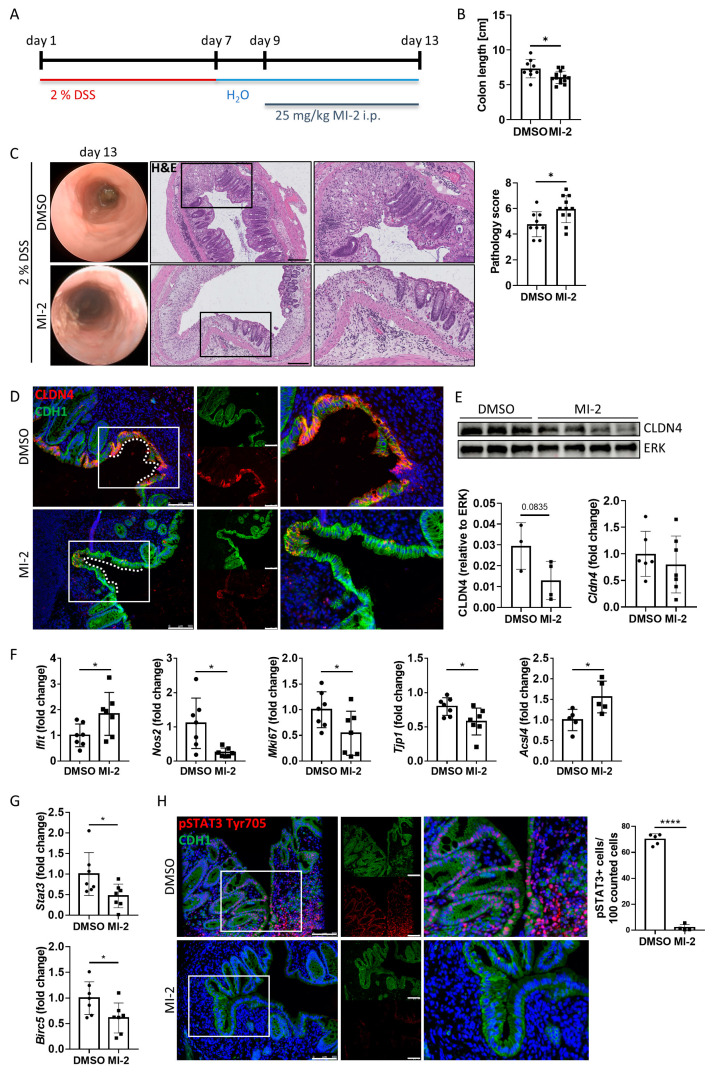
MALT1 protease mediated pSTAT3 signaling promotes mucosal wound healing in vivo. (**A**) C57BL/6J mice received 2% DSS in the drinking water for 7 days to induce acute colitis. A daily dose of 25 mg/kg MI-2 inhibited MALT1 proteolytic activity during mucosal recovery (day 9–13) (n = 13). Control mice (n = 9) were injected with DMSO. Mice were analyzed on day 13. Experiments were repeated three times with similar results. * *p* < 0.05 in unpaired two-tailed *t*-test; error bars represent mean ± SD, 95% CI. (**B**) Colon length measurement in cm. (**C**) Representative images of endoscopic analysis and H&E staining of colonic cross sections on day 13 (scale bar: 200 µm). Pathology score of endoscopic analysis. * *p* < 0.05 in unpaired two-tailed *t*-test; error bars represent mean ± SD, 95% CI. (**D**) Representative images of colonic cross sections stained immunohistochemically with antibodies against claudin-4 (red) and E-cadherin (CDH1, green), nuclei were counterstained with Hoechst 33342 (blue) (scale bar: 100 µm). (**E**) Western blot analysis and protein volume quantification of claudin-4 protein levels in whole colon tissue. ERK was used as a loading control. Quantitative real-time PCR of *Cldn4* gene expression levels. (**F**,**G**) Quantitative real-time analysis of (**F**) intestinal inflammation (DMSO n = 7, MI-2 n = 7) and ferroptosis associated genes (DMSO n = 5, MI-2 n = 5) and (**G**) wound healing associated genes (DMSO n = 7, MI-2 n = 7). * *p* < 0.05 in unpaired two-tailed *t*-test; error bars represent mean ± SD, 95% CI. (**H**) Representative colonic cross sections stained immunohistochemically with antibodies against pSTAT3 Tyr705 (red) and E-cadherin (CDH1, green), nuclei were counterstained with Hoechst 33342 (blue) (scale bar: 100 µm). Quantification of pSTAT3 positive cells in the intestinal epithelium. **** *p* < 0.0001 in unpaired two-tailed *t*-test; error bars represent mean ± SD, 95% CI.

To further study the role of the MALT1 protease in barrier function and mucosal wound healing in vivo, acute colitis was induced in C57BL/6J mice using 2% DSS. During mucosal recovery, mice were treated daily with 25 mg/kg MI-2 (Figure 6A). In line with previous results from Bornancin et al. [33] and Monajemi et al. [34], MI-2 treated mice displayed signs of persisting intestinal inflammation, which included shortened colon length and a delay in mucosal healing (Figure 6B). Further, these mice showed a higher and persistent disease activity during endoscopic analysis on day 13 (Figure 6C). This was accompanied by severe epithelial tissue destruction as shown in histological colon cross sections, confirmed by an elevated histological pathology score which includes evaluation of the integrity of the intestinal epithelium, mucosal inflammation, and changes of the submucosa (immune cell infiltration, edema formation) (Figure 6C). Wound-associated intestinal epithelial cells are characterized by expression of the tight junction protein claudin-4 (CLDN4) [35]. While *Cldn4* gene expression did not change due to MALT1 protease inhibition, we observed less CLDN4 protein localized at epithelial lesions as shown by immunofluorescence staining and a decreased CLDN4 protein level in whole colon tissue protein lysates in response to MI-2 treatment (Figure 6D,E). Consistent with our in vitro studies, this implies a potential role for MALT1 protease function in regulating epithelial tight junction biology to enhance the maintenance of the mucosal barrier and to provide intestinal homeostasis. Significantly increased colonic *Ifit1* mRNA expression as well as decreased expression levels of the proliferation marker *Mki67* and the essential mucosal wound healing factor *Tjp1* [36] supported the finding that inhibition of MALT1 protease during mucosal recovery affected cell proliferation in the intestinal epithelium, which finally delayed the wound healing process. Further, *Nos2* gene expression was significantly downregulated in response to MI-2 treatment indicating impaired NF-κB signaling in the absence of MALT1 proteolytic activity (Figure 6F). In line with our observation that MALT1 protease function can inhibit ferroptosis, MI-2 treatment in vivo was accompanied by strongly increased expression levels of pro-ferroptotic acyl-CoA synthetase long-chain family member 4 (*Acsl4*) (Figure 6F) promoting enrichment of polyunsaturated fatty acids in cellular membranes, which in turn enhances lipid peroxidation and ferroptosis [37]. This identifies the MALT1 protease as a potential negative regulator of ferroptosis in the intestinal epithelium in the context of mucosal healing. Further, interleukin (IL) 22 dependent epithelial signal transducer and activator of transduction 3 (STAT3) activation has been reported to promote mucosal wound healing [38]. *Stat3* gene expression levels were significantly downregulated in MI-2 treated mice. In line with this, the gene expression of *Birc5* (baculoviral IAP repeat-containing 5), a STAT3 target gene whose activity benefits wound healing by inducing angiogenesis [39] and has been reported to promote proliferation of LGR5^+^ stem cells and transit amplifying cells in adult intestinal tissue [40], was significantly reduced (Figure 6G). Immunofluorescence staining further revealed that epithelial STAT3 Tyr705 phosphorylation, which is associated with cell proliferation, survival and self-renewal [41], was almost absent in these mice, while in control animals about 70% of epithelial cells expressed pSTAT3 (Figure 6H). 

These data indicate a comprehensive role for the MALT1 protease in regulating mucosal wound healing during experimental colitis. As a potential negative regulator of ferroptosis in colonic tissue and regulator of wound-associated epithelial CLDN4, it supports the maintenance of the epithelial barrier and mucosal homeostasis. In addition, it mediates STAT3 activation in epithelial cells, which in turn induces mucosal healing. 

### 2.7. MALT1 Regulates STAT3-Mediated Wound Healing and Ferroptosis during Active UC

**Figure 7:**
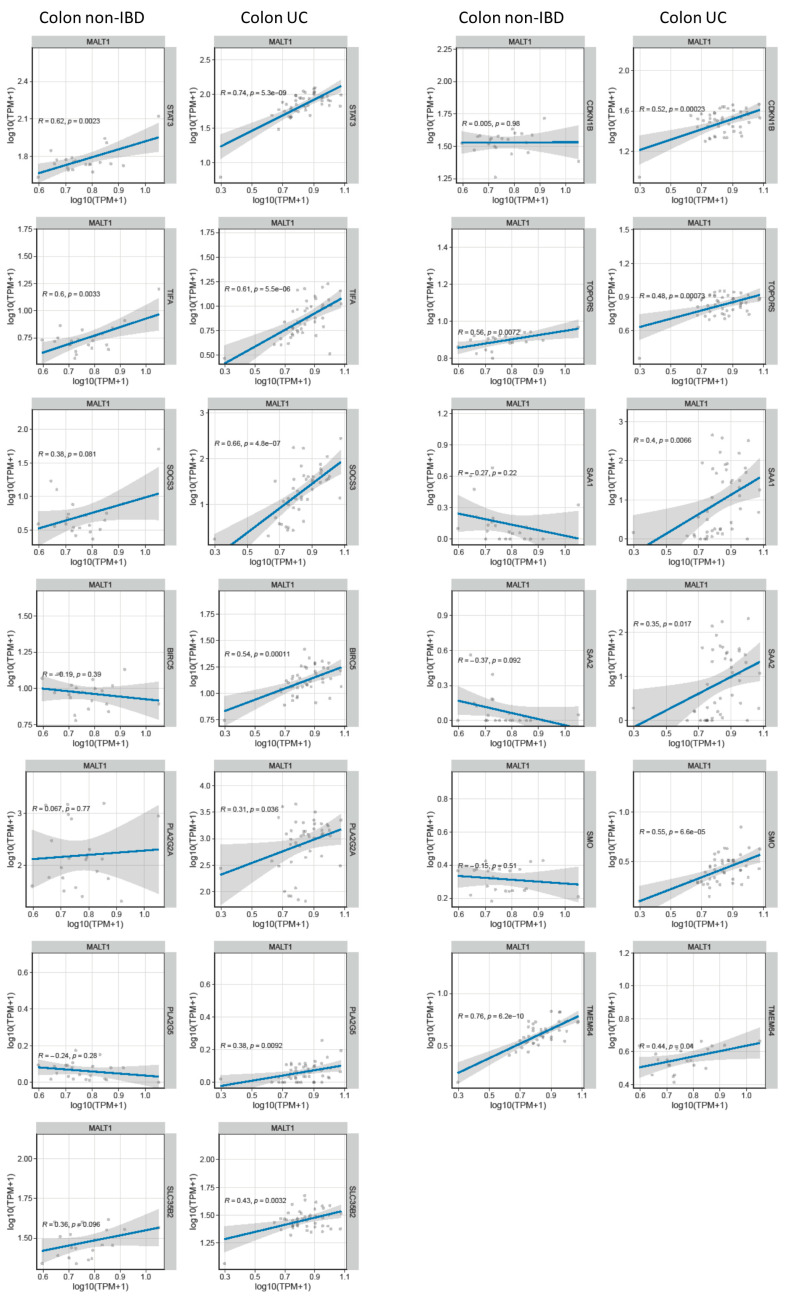
MALT1 gene expression levels correlate with wound healing associated gene expression in UC. RNA-seq correlation analysis of *MALT1* and STAT3-regulated wound healing associated genes.

Genome-wide expression analysis of DSS treated mice carrying a specific deletion of *Stat3* in the intestinal epithelium (*Stat3*^ΔIEC^) revealed a gene set that under inflammatory conditions is regulated by this transcription factor (GSE15955). Within this gene set, a wound healing and cellular stress response associated cluster was identified [38]. RNA-seq analysis of human UC colon tissue demonstrated a positive correlation of these wound healing associated and STAT3-regulated genes with *MALT1* gene expression levels during active colitis (Figure 7A). This further strengthens the hypothesis that in the context of colitis, MALT1 might promote the activation of STAT3. 

## 3. Discussion

Ulcerative colitis (UC) is a chronic inflammatory bowel disease characterized by inflammation of the colon and rectum. Mucosal healing, or the restoration of the intestinal barrier function, is an important therapeutic goal in UC, as it can improve symptoms and prevent disease progression. Disruption of the intestinal barrier can lead to the translocation of bacteria and other microorganisms across the intestinal mucosa, which can exacerbate inflammation and contribute to disease severity. Resolving inflammation and promoting mucosal healing can help to prevent this translocation and to improve outcomes in UC [42,43].

**Figure 8:**
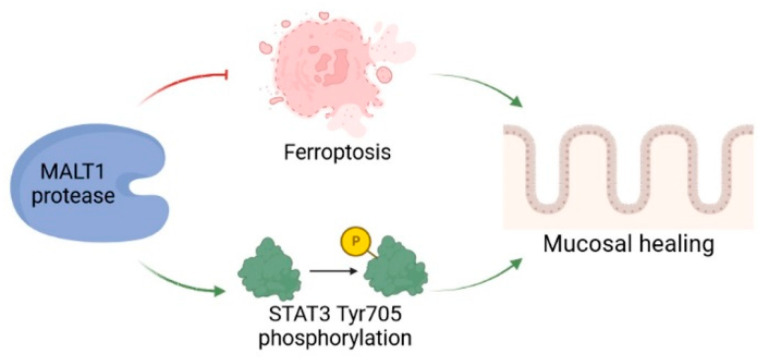
The role of MALT1 protease during mucosal wound healing. The proteolytic activity of MALT1 contributes to mucosal healing by blocking ferroptosis and promoting STAT3 activation in the intestinal epithelium. Created in Biorender.com.

We detected a significant enrichment of MALT1 gene and protein expression in the colon of UC patients, particularly in intestinal epithelial cells. Analysis of a DSS time course RNA-seq dataset further revealed increased expression of *Malt1* and the CBM complex protein *Card9* during active colitis. Together this indicates a specific contribution of MALT1 to the disease, which has also been proposed before and has identified MALT1 as a potential therapeutic target in IBD [27]. However, whether MALT1 is also involved in recovery from intestinal inflammation remains unclear. In this study we identified the MALT1 protease as a central regulator of colitis associated mucosal healing (Figure 8). 

Our data show that MALT1 proteolytic activity is necessary for the maintenance of intestinal homeostasis. It supports the intestinal barrier by regulating tight junctions and promotes epithelial cell proliferation. These properties facilitate a rapid repair of epithelial injuries which in turn has anti-inflammatory effects. The assignment of proliferative and pro-repair capacities to the MALT1 protease strongly advise against MALT1 as a therapeutic target in the context of mucosal inflammation.

MALT1 plays a key role in the activation of NF-κB signaling, a critical pathway in immune and inflammatory responses. In 2020 Wu et al. demonstrated that NF-κB regulates ferroptosis in intestinal epithelial cells. They further demonstrated that ferroptosis is involved in IEC death in the context of UC, and that ferroptosis is a potential therapeutic target for UC. Mechanistically they proved that phosphorylated p65 in the intestinal epithelium significantly inhibited ferroptosis and thus contributed to the resolution of the inflammation [6]. Here, we could further expand these analyses by demonstrating that MALT1, as an upstream regulator of NF-κB signaling, and might also control ferroptosis during intestinal inflammation. The pro-ferroptotic gene *ACSL4* has been shown to be three-fold upregulated in intestinal mucosal biopsies derived from IBD patients and was identified as a ferroptosis-related hub gene in UC, making it a key protein in disease development [44,45]. Here we show that the MALT1 protease inhibits *Acsl4* expression during recovery from experimental colitis in mice. ACSL4 promotes biosynthesis and enrichment of long chain polyunsaturated fatty acids (PUFA) in cellular membranes. As lipid peroxidation occurs more frequently at PUFAs, this facilitates ferroptosis and induces lesions in the intestinal epithelium [37]. Accordingly, MALT1 proteolytic activity negatively regulates ferroptosis by limiting *Acsl4* expression during mucosal wound healing.

*STAT3* expression has been reported to be upregulated during active inflammation and clinical remission in mucosal biopsies from UC patients, indicating a potential contribution of STAT3 to mucosal wound healing [46]. Moreover, mice lacking STAT3 in intestinal epithelial cells display a severe defect of epithelial restitution further demonstrating that intestinal epithelial STAT3 activation regulates immune homeostasis in the gut by promoting IL-22-dependent mucosal wound healing [38]. Here we demonstrate that in mice, expression and STAT3 Tyr705 phosphorylation might be regulated by MALT1 proteolytic activity during mucosal healing. Pharmacological inhibition of the MALT1 protease during recovery from experimental colitis significantly reduced *Stat3* expression levels as well as STAT3 activation in the intestinal epithelium, which in turn might have promoted the delay in mucosal healing. In addition, expression of previously identified STAT3-regulated wound healing-associated genes [38] correlated positively with *MALT1* expression in colonic tissue derived from UC patients. These data suggest that the MALT1 protease promotes STAT3-mediated wound healing during colitis.

We conclude that MALT1 protease function plays a critical role in the regulation of immune and inflammatory responses, as well as mucosal healing by inhibition of inflammation-associated ferroptosis and positive regulation of the STAT3 axis. Understanding the mechanisms by which MALT1 protease function regulates these processes may offer novel therapeutic targets for the treatment of IBD and other inflammatory diseases. Further studies are needed to determine whether it may be a therapeutic target in IBD.

## 4. Material and Methods

### 4.1. Human Samples

All studies with human material were approved by the ethics committee of the University Hospital of Erlangen (# 49_20B, 159_15 B), or the ethics committee of Charité—Universitätsmedizin Berlin (no. EA4/015/13). The diagnosis of inflammatory bowel disease was based on clinical, endoscopical and histological findings. Disease activity was evaluated based on the Harvey Bradshaw Index. 

### 4.2. Animals and Housing

*Malt1*^fl^ mice were kindly provided by R. Beyaert and were described earlier [47]. *Malt1*^ΔIEC^ mice were generated by crossing *Malt1*^fl^ mice to *Villin-Cre* mice which were described earlier [48,49]. Mice were screened regularly according to FELASA guidelines. Experimental protocols were approved by the Institutional Animal Care and Use Committee of the Regierung von Unterfranken.

### 4.3. DSS Colitis

Mice received 2% dextran sodium sulfate (DSS) (MP Biomedicals, Santa Ana, CA, USA, Cat. No. 160110) in sterilized tap water for six days to induce acute colitis followed by drinking water for nine days. From recovery day three to nine mice were daily injected intraperitoneally with 25 mg/kg MI-2 (Selleckchem, Houston, TX, USA, Cat. No. S7429) in 5% DMSO + 30% PEG-300 + 5% Tween-20 + aqua injectable.

Colitis development was monitored using a high-resolution video mini-endoscopic system. Colitis pathology was graded according to the murine endoscopic index of colitis severity (MEICS) involving evaluation of colon wall thickening, changes of the vascular pattern, fibrin visibility, granularity of the mucosal surface, and stool consistency [50].

### 4.4. Histology and Immunohistochemistry

Formalin-fixed and paraffin-embedded tissue cross sections were stained with Mayer’s haematoxylin and eosin (H&E). Immunofluorescence staining of tissue sections was performed using the TSA cyanine 3 system (Akoya Biosciences, Marlborough, MA, USA, Cat. No. SKU NEL704A001KT) according to the manufacturer’s instructions. Primary antibodies were incubated overnight at 4 °C: MALT1 (Abcam, Boston, MA, USA, Cat. No. 33921), claudin-4 (Invitrogen, Waltham, MA, USA, Cat. No. 36-4800), p65 (Cell Signaling, Danvers, MA, USA, Cat. No. 8242), pSTAT3 Tyr705 (Cell Signaling, Cat. No. 9145), E-Cadherin (BD, Franklin Lakes, NJ, USA, Cat. No. 612130). Secondary anti-rabbit (Dianova, Hamburg, Germany, Cat. No. 111-065-144) was incubated for 1 h at room temperature. Hoechst (Invitrogen, Cat. No. 33342) was used for staining nuclei. Images were taken using the confocal fluorescence LEICA TCS SP5 II microscope, or a LEICA DMI 4000B microscope together with the LEICA DFC360 FX or LEICA DFC420 C camera and the corresponding imaging software.

### 4.5. Organoid Culture

Intestinal crypts were isolated from the mouse small intestine and cultured in organoid medium supplemented with murine recombinant EGF, noggin and R-spondin for at least seven days to enable organoid formation according to Sato et al. [51]. Human tumor organoids were cultured in Advanced DMEM/F-12 (Gibco, Waltham, MA, USA, Cat. No. 12634028) supplemented with R-spondin, noggin, 1X B27, 1.25 mM N-acetylcystein, 50 µg/mL Primocin, 500 nM A83-01, 10 µM SB202190, 50 ng/mL human recombinant EGF. Murine tumor organoids (VAKPT) were cultured in Advanced DMEM/F-12 supplemented with 1X B27, 1 mM N-acetylcysteine, 50 ng/mL murine recombinant EGF and 0.5 nM dexamethasone. Organoid growth was monitored by light microscopy. Organoids were stimulated with 4 µM or 30 µM MI-2 (Selleckchem, Cat. No. S7429) and stained with propidium iodide staining solution (BD Pharmingen, San Diego, CA, USA, Cat. No. 556463). Time-lapse microscopy was performed using the EVOS M7000 Imaging System.

### 4.6. Organoid Formation Assay

Human and murine tumor organoids were dissociated into single cells in 1X TrypLE Select (Gibco, Cat. No. A12177-01) + PBS, filtered using a 40 µM cell strainer and plated in Matrigel (Corning, Corning, NY, USA, Cat. No. 356231). Single cells were treated with 4 µM MI-2 (Selleckchem, Cat. No. S7429) in organoid medium. Time-lapse microscopy was performed using the EVOS M7000 Imaging System. VAKPT organoids were kindly provided by Rene Jackstadt.

### 4.7. Cell Culture

Caco2 and HT-29 cells were cultured in DMEM (1X) + GlutaMAX (Gibco, Cat. No. 31966-021) supplemented with 1% penicillin/streptomycin (Sigma-Aldrich, St. Louis, MO, USA, Cat. No. P4333) and 10% FCS (Sigma-Aldrich, Cat. No. F7524). For Caco2-bbe cells the medium was additionally supplemented with non-essential amino acids (- L-glutamine) (Sigma-Aldrich, Cat. No. M7145) and 100 mM HEPES (Sigma-Aldrich H0887). HCT116 cells were cultured in McCoy’s 5A medium (Sigma-Aldrich, Cat. No. M4892) supplemented with 1% penicillin/streptomycin and 10% FCS. All cell lines used in this study were obtained from the American Type Culture Collection (ATCC). 

Cells were stimulated with 13.5 µM MI-2 (Selleckchem, Cat. No. S7429), 0.5 µM NSA (Merck, Darmstadt, Germany, Cat. No. 432531-71-0), 10 µM necrostatin-1 (Enzo, Farmingdale, NY, USA, Cat. No. BML-AP309), 20 µM VX-765 (Selleckchem, Cat. No. S2228), 20 µM z-VAD-FMK (MedChemExpress, Monmouth Junction, NJ, USA, Cat. No. HY-16658B), 0.2 µM liproxstatin-1 (Biomol, Hamburg, Germany, Cat. No. Cay17730) and 30 µM mepazine (MedChemExpress, Cat. No. HY-121282A). Proliferation assays were performed using the Agilent xCELLigence System or Sartorius Incucyte SX5. For Incucyte analysis cells were stained with SYTOX green nucleic acid stain (Thermo Fisher, Waltham, MA, USA, Cat. No. S7020). 

### 4.8. Immunocytochemistry

Confluent Caco2-bbe cells on transwell inserts (Millicell^®^, Cell culture inserts, 0.4 µm PCF, Merck Millipore Ltd., Carrigtwohill, Ireland; Cat. No. PIHP01250) were fixed with 1% PFA (Roth, Karlsruhe, Germany, Cat. No. 0964.2) in PBS^+Mg/Ca^, permeabilized using 0.5% Triton X 100 (Roth, Cat. No. 3051.3) in PBS^+Mg/Ca^, blocked with 6% goat serum (Sigma-Aldrich, Cat. No. G9023) + 1% BSA (Sigma-Aldrich, Cat. No. F7524) in PBS^+Mg/Ca^ and incubated with the following primary antibodies: claudin-1 (Invitrogen, Cat. No. 51-9000), claudin-2 (Invitrogen, Cat. No. 51-6100), claudin-3 (Invitrogen, Cat. No. 34-1700), claudin-4 (Invitrogen, Cat. No. 32-9400), ZO-1 (Invitrogen Cat. No. 339194), phalloidin-594 (Dyomics, Jena, Germany, Cat. No. 594-33). Goat anti-mouse (Invitrogen, Cat. No. A32728), Goat anti-mouse (Invitrogen, Cat. No. A32723) and Goat anti-rabbit (Invitrogen, Cat. No. A32731) were used as secondary antibodies. Hoechst (Invitrogen, Cat. No. 33342) was used for staining of nuclei.

### 4.9. Transepithelial Electrical Resistance (TEER) Assay

Caco2-bbe cells were seeded on transwell inserts (Millicell^®^, cell culture inserts, 0.4 µm PCF, Merck Millipore Ltd., Cat. No. PIHP01250) in cell culture medium. Confluent cells were exposed to MI-2 (Selleckchem, Cat. No. S7429) from basolateral and apical sides. To determine epithelial barrier function, TEER was measured at 37 °C using chopstick electrodes and an Ohmmeter (EVOM, WPI, Sarasota, FL, USA). The electrical resistance of the filter membrane was determined and corrected for resistance of the empty filter and the area as previously described [52].

### 4.10. Wound Healing Assay

Caco2 cells were seeded on 2-well culture dishes (ibidi, Gräfelfing, Germany, Cat. No. 81176) in culture medium. Confluent cells were kept on starving medium (culture medium without FCS) overnight. The 2-well insert was removed, the starving medium was replaced with culture medium, and cells were exposed to 2.5 µM MI-2 (Selleckchem, Cat. No. S7429). Time-lapse microscopy was performed using the EVOS M7000 Imaging System.

### 4.11. MALT1 Knockout Cells

sgRNAs were generated by cloning MALT1 specific oligomers (MALT1 CRISPR/Cas9 KO Plasmid (h2), Santa Cruz, Dallas, TX, USA, Cat. No. sc-400791-KO-2) into the pCAG-SpCas9-GFP-U6-gRNA plasmid backbone vector (addgene, Watertown, MA, USA, Cat. No. 79144). Successful cloning was confirmed by plasmid sequencing. HT-29 and HCT116 cells were transfected with Lipofectamine 2000 transfection reagent (Invitrogen, Cat. No. P/N 52887). GFP-positive cells were FACS sorted (BD, FACSAriaII) and *MALT1* knockout was confirmed by Western blot. 

### 4.12. Gene Expression

Total RNA was isolated from whole colon biopsies, small intestinal organoids, HCT116 and Caco2 cells using the NucleoSpin RNA Mini kit for RNA purification (Macherey-Nagel, Düren, Germany, Cat. No. 740955). cDNA was synthesized using the SCRIPT cDNA Synthesis Kit (Jena Bioscience, Jena, Germany; Cat. No. PCR-511). Real-time PCR analysis was performed using LightCycler 480 SYBR Green I Master (Roche, Basel, Switzerland; Cat. No. 04887352001) and specific primer assays (Table 1).

### 4.13. Immunoblotting

Whole protein lysates were isolated from colon biopsies, HCT116, Caco2 and HT-29 cells using cell lysis buffer (Cell Signaling, Cat. No. 9803) supplemented with 1 mM PMSF (Cell Signaling, Cat. No. 8553) and centrifuged at 14,000 rpm for 20 min. MiniProtean-TGX gels (4–15% polyacrylamide; Bio-Rad, Hercules, CA, USA) were used for protein separation. Separated proteins were blotted on a PVDF (Bio-Rad, Cat. No. 1704272) or nitrocellulose membrane (Bio-Rad, Cat. No. 1704270). Membranes were incubated with the following primary antibodies: MALT1 (Cell Signaling, Cat. No. 2494), ERK (Cell Signaling, Cat. No. 9102), p100/p52 (Cell Signaling, Cat. No. 4882), claudin-4 (Invitrogen, Cat. No. 36-4800). Anti-rabbit IgG, HRP-linked antibody (Cell Signaling, Cat. No. 7074) was used as a secondary antibody. Western Lightning Plus Chemiluminescent Substrate (PerkinElmer, Waltham, MA, USA, Cat. No. NEL105001EA) was used for detection. Densitometric analysis was performed using the Image Lab Software (Bio-Rad).

### 4.14. Statistical Analysis

Two groups were compared using the unpaired two-tailed *t*-test and multiple groups were compared using the ANOVA multiple comparison analysis. *p* < 0.05 (NS *p* ≥ 0.05; * *p* < 0.05; ** *p* < 0.01; *** *p* < 0.001; **** *p* < 0.0001) was considered as statistically significant. Statistical analysis was performed using GraphPad Prism 8.3.0.

## Figures and Tables

**Table 1:** Specific primer assays used for real-time PCR analysis.

Primer Assay	Company, Cat. No.
Mm_Acsl4_1_SG QuantiTect Primer Assay	Qiagen (Hilden, Germany), QT00141673
Mm_Birc5_1_SG QuantiTect Primer Assay	Qiagen, QT00113379
Mm_Cldn4_1_SG QuantiTect Primer Assay	Qiagen, QT00252084
Hs_GAPDH_1_SG QuantiTect Primer Assay	Qiagen, QT00079247
Mouse Gapdh forward (tcaccaccatggagaaggc)	Biomers (Ulm, Germany)
Mouse Gapdh reverse (gctaagcagttggtggtgca)	
Mm_Ifit1_1_SG QuantiTect Primer Assay	Qiagen, QT01161286
Hs_MALT1_1_SG QuantiTect Primer Assay	Qiagen, QT00032718
Mm_Mki67_1_SG QuantiTect Primer Assay	Qiagen, QT00247667
Mm_Nos2_1_SG QuantiTect Primer Assay	Qiagen, QT00100275
Mm_Stat3_1_SG QuantiTect Primer Assay	Qiagen, QT00148750
Mm_Tjp1_1_SG QuantiTect Primer Assay	Qiagen, QT00493899

## Data Availability

The RNA-seq datasets used in this study have been deposited in the Array Express service of the Molecular Biology Laboratory–European Bioinformatics Institute under the accession IDs E-MTAB-9850 (DSS time course) [53] and E-MTAB-10824 (wound healing time course) [35].

## Supplementary Materials

The supporting information can be downloaded at https://www.mdpi.com/article/10.3390/ijms24087402/s1.
