# Supplementary material for: Proteolytic Activity of the Paracaspase MALT1 Is Involved in Epithelial Restitution and Mucosal Healing
Source: Int J Mol Sci. 2023 Apr 17;24(8):7402. doi: 10.3390/ijms24087402 (PMC10138456; doi:10.3390/ijms24087402)
Supplement: Supplementary file 1 [file ijms-24-07402-s001.zip › ijms-2298806-supplementary.pdf]

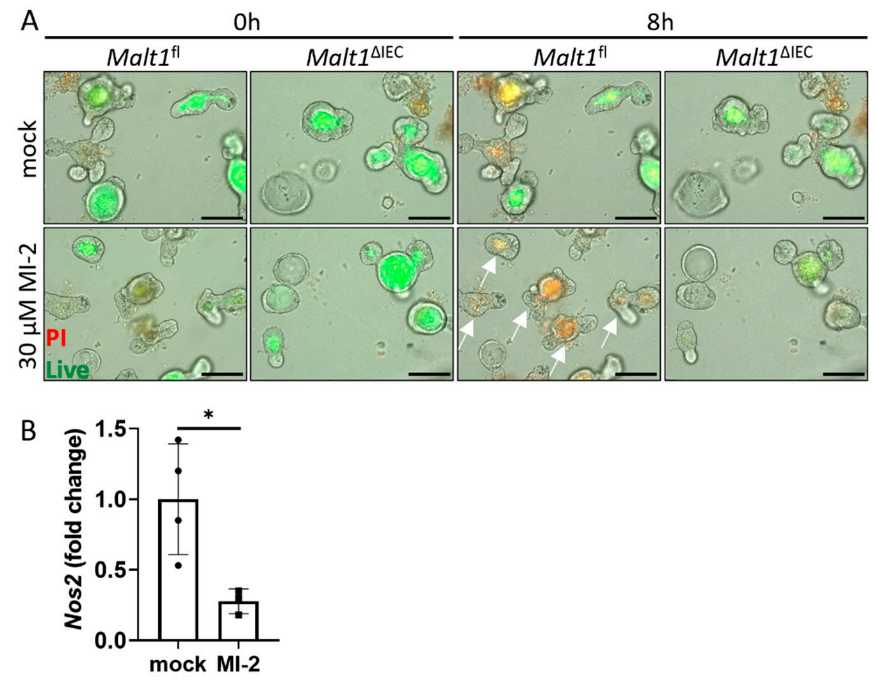

Figure S1

Figure S1. (**A**) Small intestinal organoids derived from control (*Malt1*^{fl}) and *Malt1*$^{\Delta IEC}$ mice were stimulated with 30 µM MI-2 for 8 h. Organoid survival was monitored by propidium iodide (PI) staining (red, dead cells) and green autofluorescence of live cells, arrows indicate dying organoids (scale bar: 500 µm). (**B**) Quantitative real-time PCR of *Nos2* gene expression levels in wildtype murine small intestinal organoids stimulated with 30 µM MI-2 for 4 h.
